# A structural genomics approach to investigate Dystrophin mutations and their impact on the molecular pathways of Duchenne muscular dystrophy

**DOI:** 10.3389/fgene.2025.1517707

**Published:** 2025-02-04

**Authors:** Abdelbaset Mohamed Elasbali, Farah Anjum, Osama A. AlKhamees, Waleed Abu Al-Soud, Mohd Adnan, Anas Shamsi, Md. Imtaiyaz Hassan

**Affiliations:** ^1^ Department of Clinical Laboratory Science, College of Applied Medical Sciences-Qurayyat, Jouf University, Sakakah, Saudi Arabia; ^2^ Department of Clinical Laboratory Sciences, College of Applied Medical Sciences, Taif University, Taif, Saudi Arabia; ^3^ Department of Pharmacology, College of Medicine, Imam Mohammad Ibn Saud Islamic University (IMSIU), Riyadh, Saudi Arabia; ^4^ Molekylärbiologi, Klinisk Mikrobiologi och vårdhygien, Lund, Sweden; ^5^ Department of Biology, College of Science, University of Ha’il, Ha’il, Saudi Arabia; ^6^ Centre of Medical and Bio-Allied Health Sciences Research, Ajman University, Ajman, United Arab Emirates; ^7^ Centre for Interdisciplinary Research in Basic Sciences, Jamia Millia Islamia, New Delhi, India

**Keywords:** deleterious mutations, Duchenne muscular dystrophy, Dystrophin, neuromuscular diseases, personalized medicine, structural genomics

## Abstract

**Background:**

Dystrophin is a key protein encoded by the *DMD* gene, serves as a scaffold linking the cytoskeleton to the extracellular matrix that plays a critical role in muscle contraction, relaxation, and structural integrity. Mutations, particularly single-point amino acid substitutions, can lead to dysfunctional Dystrophin, causing muscular dystrophies, with Duchenne muscular dystrophy (DMD) being the most severe form.

**Objective:**

This study aimed to evaluate the effects of 184 single-point amino acid substitutions on the structure and function of Dystrophin using computational approaches.

**Methods:**

Many computational tools were used to predict the impact of amino acid substitutions on protein stability, solubility, and function. Pathogenic potential was assessed using disease phenotype predictors and CADD scores, while allele frequency data from gnomAD contextualized mutation prevalence. Additionally, aggregation propensity, frustration analysis, and post-translational modification sites were analyzed for functional disruptions.

**Results:**

Of the 184 substitutions analyzed, 50 were identified as deleterious, with 41 predicted to be pathogenic. Seventeen mutations were localized in the Calponin-homology (CH) 1 domain, a critical functional region of Dystrophin. Six substitutions (N26H, N26K, G47W, D98G, G109A, and G109R) were predicted to decrease protein solubility and were located in minimally frustrated regions, potentially compromising Dystrophin functionality and contributing to DMD pathogenesis.

**Conclusion:**

This study provides novel insights into the molecular mechanisms of DMD, highlighting specific mutations that disrupt Dystrophin’s solubility and function. These findings could inform future therapeutic strategies targeting Dystrophin mutations to address DMD pathogenesis.

## Introduction

Duchenne muscular dystrophy (DMD) is among the most severe and widespread types of muscular dystrophy that affects approximately 1 in every 5,000 male births worldwide (Ke et al., 2019). This is an X-linked recessive disorder that primarily occurs in early childhood and results in premature mortality, typically by the third decade of life (Younger, 2023). Central to the pathogenesis of DMD is the disruption of Dystrophin, a critical protein encoded by the *DMD* gene located on the X chromosome (Brown and Hoffman, 1988). Dystrophin plays a central role in maintaining the structural integrity and functionality of muscle fibers (Gao and McNally, 2015). It serves as a scaffold protein that links the internal cytoskeleton of muscle cells with the extracellular matrix and offers crucial structural support throughout muscle contraction and relaxation (Wilson et al., 2022). Mutations in the *DMD* gene result in a defective or nonfunctional Dystrophin protein, which causes a group of muscle-wasting disorders known as muscular dystrophies (Duan et al., 2021). Of these, DMD is the most severe phenotype, characterized by the dysfunction of Dystrophin expression (Bonilla et al., 1988).

Over the years, the identification of single-point amino acid substitutions in Dystrophin has been markedly increased (Roberts et al., 1992; Muntoni et al., 2003; Fortunato et al., 2021). However, interpreting the functional consequences of these substitutions and elucidating their precise roles in DMD pathogenesis remain tough challenges (Fuller et al., 2016). The diversity and complexity of Dystrophin mutations require a thorough analysis to evaluate their effects on protein structure, function, and, ultimately, disease progression (Fuller et al., 2016). In recent years, bioinformatics methods have emerged as helpful tools to predict the functional impact of genetic variants, including single-point amino acid substitutions, in disease-associated genes and gene products (Tang and Thomas, 2016; Amir et al., 2019a; Umair et al., 2021). In this context, bioinformatics algorithms and structural modeling techniques can systematically evaluate the pathogenic potential of Dystrophin mutations and prioritize variants for further experimental validation. Moreover, computational analyses can offer deeper insights into the molecular mechanisms underlying DMD that can guide the development of targeted therapeutic interventions and personalized treatment strategies.

In this study, we performed a detailed analysis of deleterious single-point amino acid substitutions in Dystrophin and their impact on DMD pathogenesis. We employed advanced computational methods, such as SIFT (Shihab et al., 2013), PolyPhen-2 (Adzhubei et al., 2013), FATHMM (Rogers et al., 2018), SNPs&Go (Pires et al., 2014) mCSM (Laimer et al., 2015), DynaMut2 (Capriotti et al., 2006), MAESTROweb (Laimer et al., 2016), PremPS (Rodrigues et al., 2021), MutPred2 (Pejaver et al., 2020), and PhD-SNP (Calabrese et al., 2008), and systematically evaluate 184 amino acid substitutions to assess their effects on Dystrophin structure and function.

To gain insights into the molecular mechanism of DMD, we focused on a group of high-probability mutations that would likely have severe effects on Dystrophin function. In conclusion, the study is powerful evidence of the need to incorporate computational analysis in the discovery of Dystrophin mutations and their involvement in DMD. The findings highlighted that such investigations may help advance the development of personalized treatments for this fatal muscle disease and thereby enhance the quality of life for patients with DMD.

## Materials and methods

### Retrieval of data

The protein sequence of human Dystrophin was taken from the UniProt protein database (Accession ID: P11532). A compilation of individual amino acid substitutions in Dystrophin was assembled using the data accessible through dbSNP (https://www.ncbi.nlm.nih.gov/snp/) (Sherry et al., 2001), Ensembl (https://asia.ensembl.org/index.html, Ensembl gene IDENSG00000198947.18) (Hubbard et al., 2002), and PubMed (https://pubmed.ncbi.nlm.nih.gov/) literature (Supplementary Data). A total of 184 mutations were taken from these sources. Duplicates were eliminated from the list of mutations. Population frequency data for these mutations were retrieved from the gnomAD database (https://gnomad.broadinstitute.org/, Genome buildGRCh38/hg38) to provide insights into their prevalence in the general population and specific cohorts. The three-dimensional structure of human Dystrophin (Actin-binding domain) was downloaded from the RCSB Protein Data Bank (PDB ID: 1DXX) (Berman et al., 2000). These datasets served as the foundation for employing various state-of-the-art computational tools, detailed in the subsequent sections, to predict the structural and functional impact of these mutations.

### Sequence-based predictions

#### PolyPhen2

PolyPhen-2 (Polymorphism Phenotyping v2) is a bioinformatics software tool used to predict the impacts of amino acid changes on protein structure and function (Ramensky et al., 2002). It assesses amino acid substitutions by considering the relative and spatial properties of the amino acids, thus approximating the chances of these substitutions altering the native conformation of the protein. PolyPhen-2 uses sequence, structural, and evolutionary information to predict the physiological relevance of amino acid changes. It employs a set of machine learning algorithms that are trained on a database of known polymorphisms to sort variants into various groups based on their potential effects, which can be labeled as “benign”, “possibly damaging”, or “probably damaging”.

#### SIFT

SIFT (Sorting Intolerant From Tolerant) is another bioinformatics tool widely applied in computational biology and genetics to estimate whether an amino acid change in a protein will be tolerated or not. Like PolyPhen-2, SIFT works to rank genetic variants based on their level of tolerance to change and their impact on protein structure and function. It aligns the amino acid at the position of interest with the amino acid residues of a homologous protein from other species. SIFT assigns a score to each amino acid substitution, with lower scores indicating a higher likelihood of being deleterious. Mutations are categorized as intolerable if the SIFT score is 0.05 or lower (Kumar et al., 2009; Ng and Henikoff, 2003).

#### FATHMM

FATHMM is an application that is employed in functional analysis with the help of hidden Markov models to predict the effects of genetic variants, especially those occurring in coding regions of the genome. It uses Hidden Markov Model (HMM) to predict the effects of variants on protein structure and function based on the data obtained from the PDB (Shihab et al., 2013). FATHMM uses features such as evolutionary conservation, physicochemical properties of the amino acid, and genomic context to rate variants. Incorporation of these features into its model allows FATHMM to output a prediction of the functional consequence of the given variant. The result of FATHMM usually includes a score or risk assessment of a genetic variant being pathogenic or benign. A high FATHMM score indicates that the variant is likely to be benign and the cells are very unlikely to be affected by it, whereas a low score indicates that the variant may have functional consequences and could be pathogenic.

#### SNPs&GO

SNPs&GO is a web server that employs an SVM for identifying deleterious single-point amino acid substitutions (Capriotti et al., 2013). The SVM classifier combines protein sequence, profile, and functional data to differentiate between disease-associated and neutral variants, utilizing gene ontology (GO) annotations. SNPs&GO employs machine learning algorithms trained on various features derived from protein sequence and structure to predict the impact of SNPs. These features include amino acid physicochemical properties, evolutionary conservation, protein domain information, and structural annotations. An SNPs&GO score surpassing 0.5 signifies a substitution that is probable to induce disease. Moreover, the tool yields output from additional resources such as PANTHER and PhD-SNP.

### Structure-based predictions

#### mCSM

mCSM (Mutation Cutoff Scanning Matrix) is a computational tool used in structural bioinformatics to predict the effects of mutations on protein stability. It is a tool designed for assessing single-point amino acid substitutions via a graph-based method (Pires et al., 2014). The predictive models are developed using environmental data extracted from atomic distance patterns of diverse residues. This tool enriches our comprehension of mutations related to diseases across a spectrum of proteins. A mCSM score (ΔΔ*G*) below 0 suggests that a mutation profoundly influences the protein structure.

#### MAESTROweb

MAESTROweb (https://pbwww.services.came.sbg.ac.at/maestro/web) is an online platform for protein structure-based prediction of the effects of mutations. MAESTROweb is especially helpful in the identification of the impact of mutations on protein stability, which is highly important for the assessment of the functional consequences of genetic changes, particularly in the context of human diseases. It uses several computational tools to evaluate the effects of mutations on the stability of proteins. MAESTROweb employs a range of computational tools and strategies, such as machine learning and structural bioinformatics, to predict the functional impact of mutations. A score of below 0 means that the protein is expected to change stability due to the amino acid substitutions (Laimer et al., 2015).

#### PremPS

PremPS (https://lilab.jysw.suda.edu.cn/research/PremPS/) assesses the impact of amino acid substitutions in proteins (Chen et al., 2020). It uses multiple approaches, such as multiple sequence alignment (MSA), protein structure, and a deep learning model, to make predictions about the potential impact of genetic variants on protein function and disease. It is specifically designed to predict whether a single amino acid substitution is likely to be deleterious or tolerated based on the protein’s sequence information. We input the amino acid sequence of the protein and specify the position and the amino acid substitution. Then, PremPS calculates a prediction score indicating the likelihood of the substitution being deleterious or tolerated.

#### DynaMut2

DynaMut2 (http://biosig.unimelb.edu.au/dynamut2/) is also a predictive tool tailored for estimating protein stability (Rodrigues et al., 2021). Amino acid substitution data were obtained from the ProTherm database. DynaMut2 can make predictions for single and multiple mutations; however, the experiment conducted on DynaMut2 focuses on single mutation prediction. DynaMut2 offers users the predicted effects of the amino acid substitution on stability and the dynamic properties of the protein, as well as the graphical representation of the mutations, to help users better understand the structural context of the changes.

### Pathogenicity prediction

#### PhD-SNP

The PhD-SNP (https://snps.biofold.org/phd-snp/phd-snp.html) is a web-based pathogenicity analysis tool that employs an SVM-based classifier to categorize variants associated with diseases (Capriotti et al., 2006). In this process, both sequence and profile information are utilized to establish a distinction between neutral and disease-associated amino acid substitutions. It employs machine learning algorithms trained on various sequence, structural, and functional features to make predictions about the functional consequences of SNPs. A PhD-SNP score exceeding 0.5 signifies an amino acid substitution likely to induce disease. We used PhD-SNP to examine the pathogenicity of mutations in Dystrophin.

#### MutPred2

MutPred2 (http://mutpred.mutdb.org) is also a web-based tool designed to classify amino acid substitutions as either disease-associated or neutral. The tool is designed to accurately estimate the likelihood that a particular amino acid substitution is likely to be deleterious or neutral and offers information about the possible molecular processes that may underlie the predicted outcomes (Pejaver et al., 2020) MutPred2 considers the sequence and structural properties of the mutated protein, such as evolutionary conservation, physicochemical properties of the amino acid substitution, protein domains, and structural annotations. Any MutPred2 score greater than 0.5 denotes a substitution considered to be pathogenic. We utilized MutPred2 to predict the pathogenicity of the mutations in Dystrophin.

#### Combined annotation dependent depletion (CADD)

The CADD (https://cadd.gs.washington.edu/) tool is used to score the deleteriousness of insertion/deletion variants, multi-nucleotide substitutions, and single nucleotide variants in the human genome. By integrating multiple annotation features, CADD evaluates both coding and non-coding variants, providing a comprehensive score that reflects the likelihood of a variant impacting biological function and contributing to disease. This score combines information from evolutionary conservation, functional genomics, and experimental data, allowing researchers to distinguish between benign and pathogenic variants. CADD was used on the screened mutations in Dystrophin.

### Aggregation propensity analysis

Aggregation propensity analysis of proteins is useful in investigating how mutations in a protein can affect its tendency to form aggregates. SODA (http://protein.bio.unipd.it/soda/) is a bioinformatics tool and web server designed to predict the solubility of proteins based on their intrinsic disorder and aggregation propensity (Paladin et al., 2017). The input file for this tool can be either a FASTA sequence or a PDB structure file. SODA predicts various types of variations while utilizing multiple algorithms such as PASTA 2.0, ESpritz-NMR, and Fells. It provides final results based on the disparity in solubility between the wild-type and mutant protein. We used SODA to evaluate the aggregation propensity of mutations in Dystrophin.

### Analysis of conserved residues

The concept of conservation of amino acids is crucial in comprehending the evolution and the structure and function of proteins. The ConSurf database (https://consurf.tau.ac.il/) is utilized to evaluate the conservation levels of residues at specific positions through multiple sequence alignment (Ashkenazy et al., 2016). ConSurf calculates the conservation score for each residue using the maximum likelihood (ML) method or empirical Bayesian method. The ConSurf scores for residues are determined according to the levels of conservation; the least conserved residues are given a score of 1, while intermediate conservation is given a score of 5, and highly conserved residues receive a score of 9. Scores for well-characterized PDB structures have been pre-computed and are available in the ConSurf-DB.

### Residual frustration analysis

Residual frustration analysis in proteins is a valuable approach to exploring the level of frustration present in the structure of a protein (Ferreiro et al., 2014). The Frustratometer server (http://frustratometer.qb.fcen.uba.ar/) was employed to assess the enduring frustration within the Dystrophin structure. We have calculated both the individual and configurational residual indices for the structure. The Frustratometer evaluates the energy of a protein structure by comparing it to a set of “decoy” states (Jenik et al., 2012). The residual frustration index between amino acids *i* and *j* is determined as a Z-score, comparing the energy of the native contact to that of *N* decoys. A contact is considered highly frustrating or destabilizing if its Z-score is below 0.78. Conversely, a contact was classified as minimally frustrated or stabilizing if the Z-score value was >0.78. Contacts falling between these thresholds were considered neutral.

### Analysis of protein-protein interaction

**FIGURE 1:**
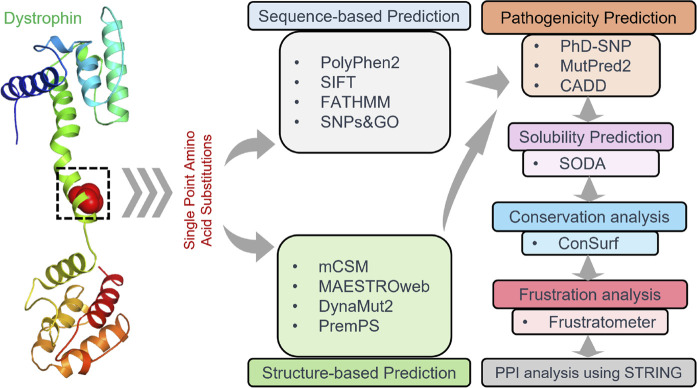
Visualization depicting the workflow pipeline of analyzing Dystrophin mutations in this study.

It is crucial to consider protein-protein interactions to understand better cellular processes and interactions between different proteins, especially in the case of the influence of pathological proteins and their connections with diseases (Mohammad et al., 2022; Mushtaq et al., 2023). The STRING database was employed to analyze the PPI network of Dystrophin as a hub. The interaction networks for Dystrophin were generated with a high confidence of 0.700 (Szklarczyk et al., 2021). Additionally, the 3D structures of the interacting proteins were obtained from SWISS-MODEL (https://swissmodel.expasy.org) to analyze these interactions further. The work pipeline employed in this study is visually depicted in Figure 1.

## Result and discussion

**FIGURE 2:**
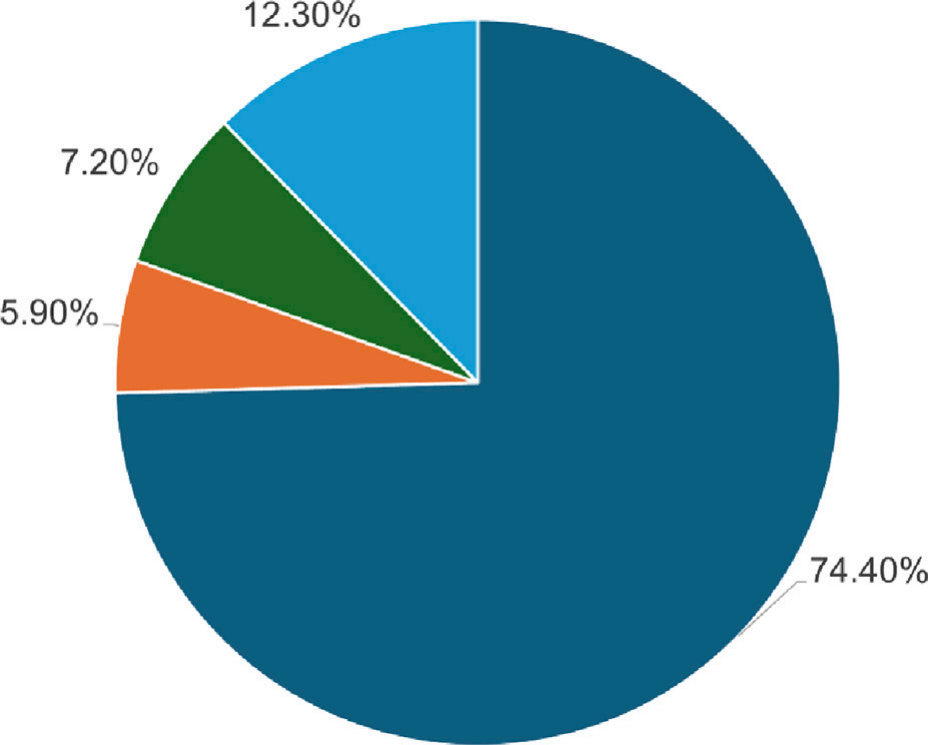
Depiction of the SNPs found within the *DMD* gene utilizing the dbSNP database.

A total of 184 single-point amino acid substitutions were sourced from the dbSNP (http://www.ncbi.nlm.nih.gov/snp) and Ensembl (http://www.ensembl.org/) databases, supplemented by mutations retrieved from the literature available on PubMed (Figure 2). We were particularly interested in determining the structural and functional consequences of these substitutions in the Dystrophin protein, which we achieved using a tiered approach. Sequence and structure information were used in the analyses to pinpoint high-confidence deleterious mutations. The sequence-based analysis utilized four web tools: SIFT, PolyPhen2, FATHMM, and SNPs&GO, which are some of the tools that can be used to predict the impact of a mutation. On the other hand, the structure-based approach used mCSM, DynaMut2, MAESTROweb, and PremPS tools to study the effects of single-point amino acid substitutions in the actin-binding region of the Dystrophin protein. To minimize the number of false positives, only those substitutions that were high-confidence mutations were pursued for further analysis. To identify diseases related to these high-confidence mutations, the PhD-SNP and MutPred2 web tools were used.

### Deleterious mutations from sequence and structure-based approaches

The utilization of multiple prediction tools in the sequence-based approach serves to mitigate false positive results and bolster the accuracy of mutation predictions. Among these tools, SIFT evaluates protein physical properties to classify mutations as either tolerated or intolerant, with a higher tolerance index indicating lower functional impact and *vice versa*. PolyPhen2 similarly employs amino acid sequences to categorize non-synonymous mutations into possibly damaging, probably damaging, or benign categories based on specific scores. To further enhance confidence levels, the inclusion of FATHMM and SNPs&GO tools strengthens the predictive capacity of the analysis. Mutations associated with diseases frequently affect the stability of proteins. Proteins can exist in folded or unfolded states. In thermodynamics, Gibbs free energy between the folded (*G*f) and unfolded (*G*u) states of a protein is determined as Δ*G* = *G*u − *G*f. The alteration in protein stability and the free energy landscape is assessed by ΔΔ*G* = *G*m − *G*w, with Gm representing the mutant protein and *G*w denoting the wild-type protein. A ΔΔ*G* value that is negative implies a mutation that stabilizes, whereas a positive ΔΔ*G* value suggests mutations that destabilize.

In this study, we utilized four different structure-based prediction tools: mCSM, DynaMut2, MAESTROweb, and PremPS. These are some of the tools that have been developed to aid in the study of protein stability and mutations. These tools take the PDB file of the wild-type protein as input and analyze atomic coordinates to predict the stability of variants using folding free energy prediction. Most of these tools follow a machine learning approach, integrating various biophysics-based methods to predict the effect of mutations on protein stability. Using this set of tools, we endeavored to offer a detailed evaluation of the structural effects of mutations within the protein of focus.

**FIGURE 3:**
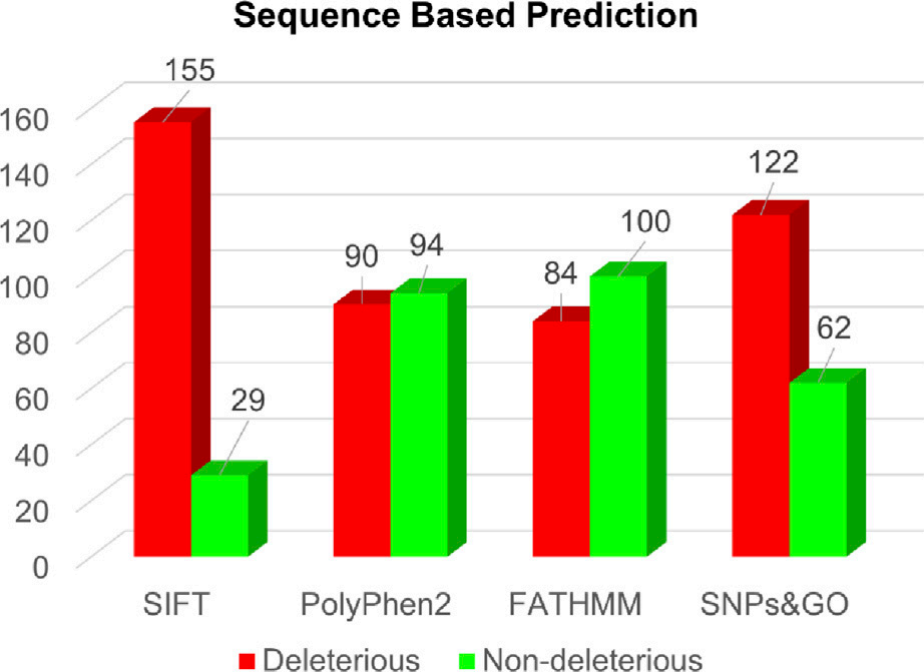
Sequence-based prediction of deleterious mutations in Dystrophin.

In the sequence-based approach, analysis of all 184 single-point amino acid substitutions within the actin-binding region of the human Dystrophin protein revealed predictions from SIFT, PolyPhen2, FATHMM, and SNPs&GO (Supplementary Table S1). Specifically, these respective tools predicted deleterious substitutions for 155, 90, 84, and 122 substitutions (Figure 3).

**FIGURE 4:**
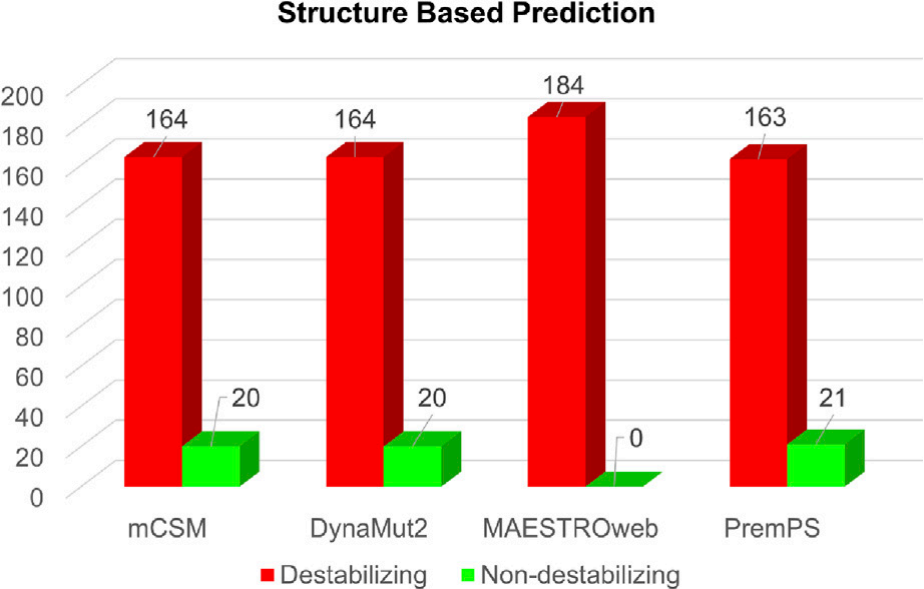
Structure-based prediction of destabilizing mutations in Dystrophin.

Simultaneously, the structure-based predictions from mCSM, DynaMut2, MAESTROweb, and PremPS identified 164, 164, 184, and 163 substitutions as destabilizing mutations (Figure 4). To enhance confidence, only mutations predicted as deleterious by all sequence-based and structure-based tools were selected for further analysis. This stringent filtering process yielded 50 amino acid substitutions predicted as both deleterious and destabilizing (Supplementary Table S2). Subsequently, these 50 substitutions were subjected to analysis for their association with disease phenotypes.

### Identification of disease-associated mutations

**FIGURE 5:**
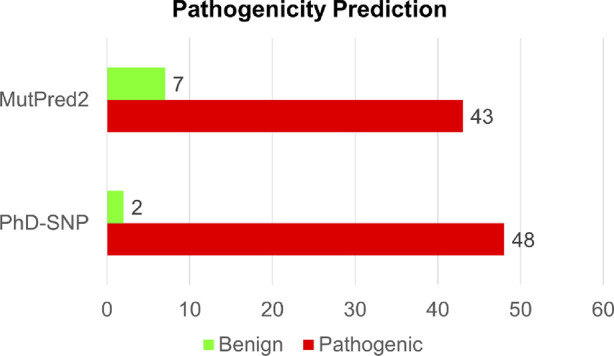
Structure-based prediction of pathogenic mutations in Dystrophin.

Analyzing disease-associated mutations in proteins is a crucial aspect of understanding the molecular basis of various complex diseases (Gao et al., 2015). In our analysis of disease-associated single-point mutations, we employed PhD-SNP and MutPred2. These methods classify mutations based on their pathogenicity scores and identify associated disease phenotypes. Among the 50 high-confidence mutations identified through both structure-based and sequence-based analyses, PhD-SNP predicted 48 substitutions as pathogenic, while MutPred2 identified 43 mutations as pathogenic (Supplementary Table S3). However, only 41 mutations were identified as pathogenic by both disease phenotype prediction tools out of the 50 mutations analyzed (Figure 5). This overlapping subset of mutations represents those with a higher likelihood of being associated with disease phenotypes, as indicated by the consensus prediction from both tools. After we arrived at the pathogenic single-point mutation, we focused on the Calponin-homology (CH) 1 domain of the actin-binding region of Dystrophin. We have removed the mutations which were present in the unstructured region of the domain. We have found 17 single amino acid substitutions destabilizing in the CH1 domain.

We have also analyzed the 50 single-point mutations using the CADD tool (Supplementary Table S3). The integration of CADD scores provided a quantitative framework to assess the deleteriousness of mutations beyond sequence and structural analyses. Among the 50 high-confidence mutations, CADD identified 43 with scores above 20, indicating likely pathogenicity. The allele frequency of the selected mutations was retrieved using gnomAD (Supplementary Table S4). Allele frequency data from the gnomAD database highlighted the rarity of 15 mutations among the general population, supporting their potential role in disease pathogenesis. This context is crucial for distinguishing rare pathogenic variants from benign polymorphisms.

### Analysis of aggregation propensity

Further, we have predicted the aggregation propensity of the protein caused by these mutations. The solubility of a protein significantly impacts its functionality (Paladin et al., 2017). The insoluble regions of a protein tend to aggregate, potentially contributing to disease progression. SODA was used to evaluate the solubility of protein variants and identify their association with disease. SODA evaluates the aggregation, disorder, helix, and strand tendencies resulting from mutations. Out of the 17 deleterious single-point amino acid substitutions obtained from disease phenotype prediction, 6 substitutions (N26H, N26K, G47W, D98G, G109A, and G109R) decrease the solubility of the protein (Table 1).

**TABLE 1:** Predicted aggregated mutants of Dystrophin protein using SODA server.

S. No.	Mutation	Helix	Strand	Aggregation	Disorder	SODA	Solubility
1	K18N	−1.135	0.18	11.268	−0.01	9.332	More soluble
2	F21S	−2.568	0.805	21.438	0.23	18.419	More soluble
3	T22K	0.942	−0.304	14.032	0.036	15.421	More soluble
4	N26D	−0.234	0.079	3.602	0.019	3.329	More soluble
5	N26H	−0.269	0.138	−15.593	0.041	−15.776	Less soluble
6	N26K	0.333	−0.145	−13.193	0.019	−12.773	Less soluble
7	G47W	0.971	−0.328	−4.736	−0.265	−3.975	Less soluble
8	L50H	−1.886	0.886	2.687	0.223	1.228	More soluble
9	L51P	−4.48	1.179	9.802	0.897	5.068	More soluble
10	L57P	−4.208	1.622	5.441	1.769	3.879	More soluble
11	V77D	−8.631	5	197.696	0.283	191.105	More soluble
12	L81Q	−2.153	1.157	178.668	0.139	177.054	More soluble
13	D98G	−0.703	0.554	−17.877	−0.003	−18.224	Less soluble
14	L108P	−4.291	1.489	507	−0.009	501.453	More soluble
15	G109A	2.015	−1.437	−106.767	0.002	−105.638	Less soluble
16	G109D	0.281	−0.577	18.496	−0.043	17.859	More soluble
17	G109R	1.311	−0.932	−22.194	−0.041	−21.462	Less soluble

### Analysis of evolutionarily conserved residues

**FIGURE 6:**
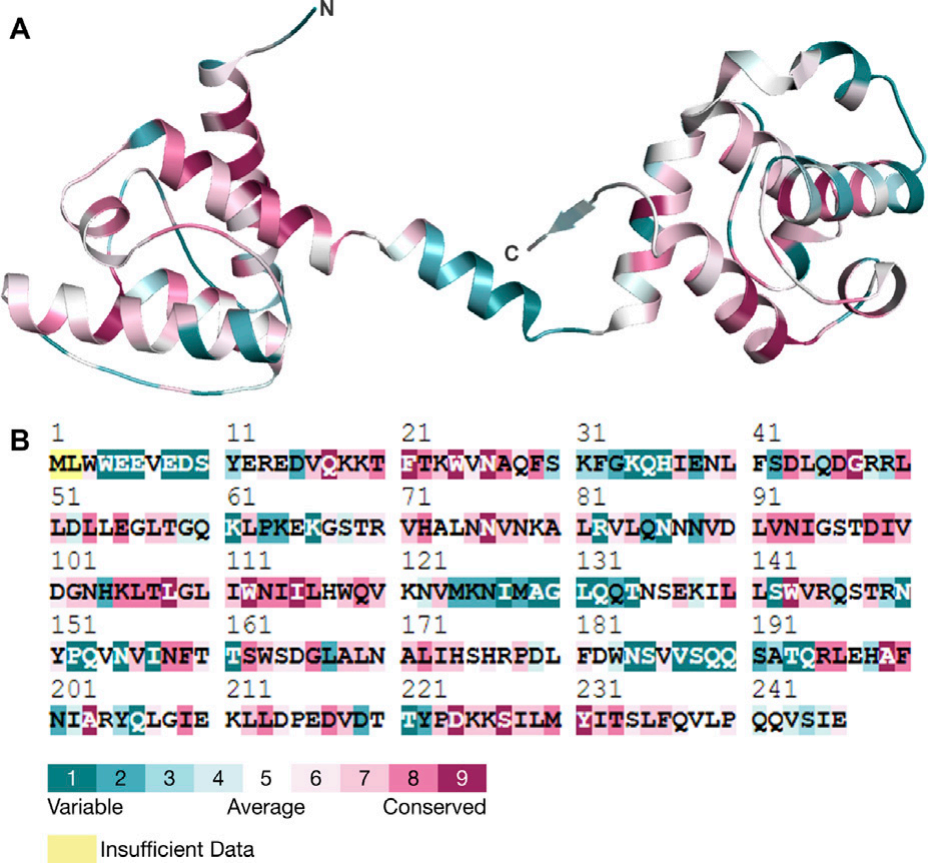
Conservation analysis. **(A)** Three-dimensional structure of Dystrophin and its residual conservation. **(B)** ConSurf plot of evolutionary conserved residues in Dystrophin.

Conservation of amino acid residues within a protein structure helps to identify the role of residues and demonstrates regional trends in evolution (Umair et al., 2021; Amir et al., 2019b). Conserved residues are important in ensuring that a protein has the right shape and structure (Kragelund et al., 1999). The conservation of an amino acid also determines the probability of a mutation occurring in the same amino acid (Guo et al., 2004). For the present study, we employed the ConSurf tool to investigate the conservation of residues in the human Dystrophin protein. It was also observed that the various residues, including N26, G47, D98, and G109, have higher conservation levels than other areas (Figure 6). This, therefore, implies that these residues are essential for the proper functioning of Dystrophin. Specifically, N26, G47, and D98 in the N-terminal of Dystrophin’s actin-binding domain had the highest conservation scores and a propensity for forming aggregated protein. This suggests that mutations in these conserved residues could greatly impact the protein and its stability and function and may lead to aggregation, which is associated with diseases. In conclusion, the evolutionary conservation analysis reveals that residues N26, G47, D98, and G109 are crucial to the structural and functional stability of the Dystrophin protein. We have also predicted the post-translational modification (PTM) sites of the protein (Supplementary Table S5). The PTM sites were predicted using MusiteDeep, which uses sequence-based and structural features to identify potential modification sites. PTM predictions revealed potential disruptions in phosphorylation and acetylation sites, which are critical for Dystrophin’s stability and interactions with other proteins.

### Residual frustration analysis

**FIGURE 7:**
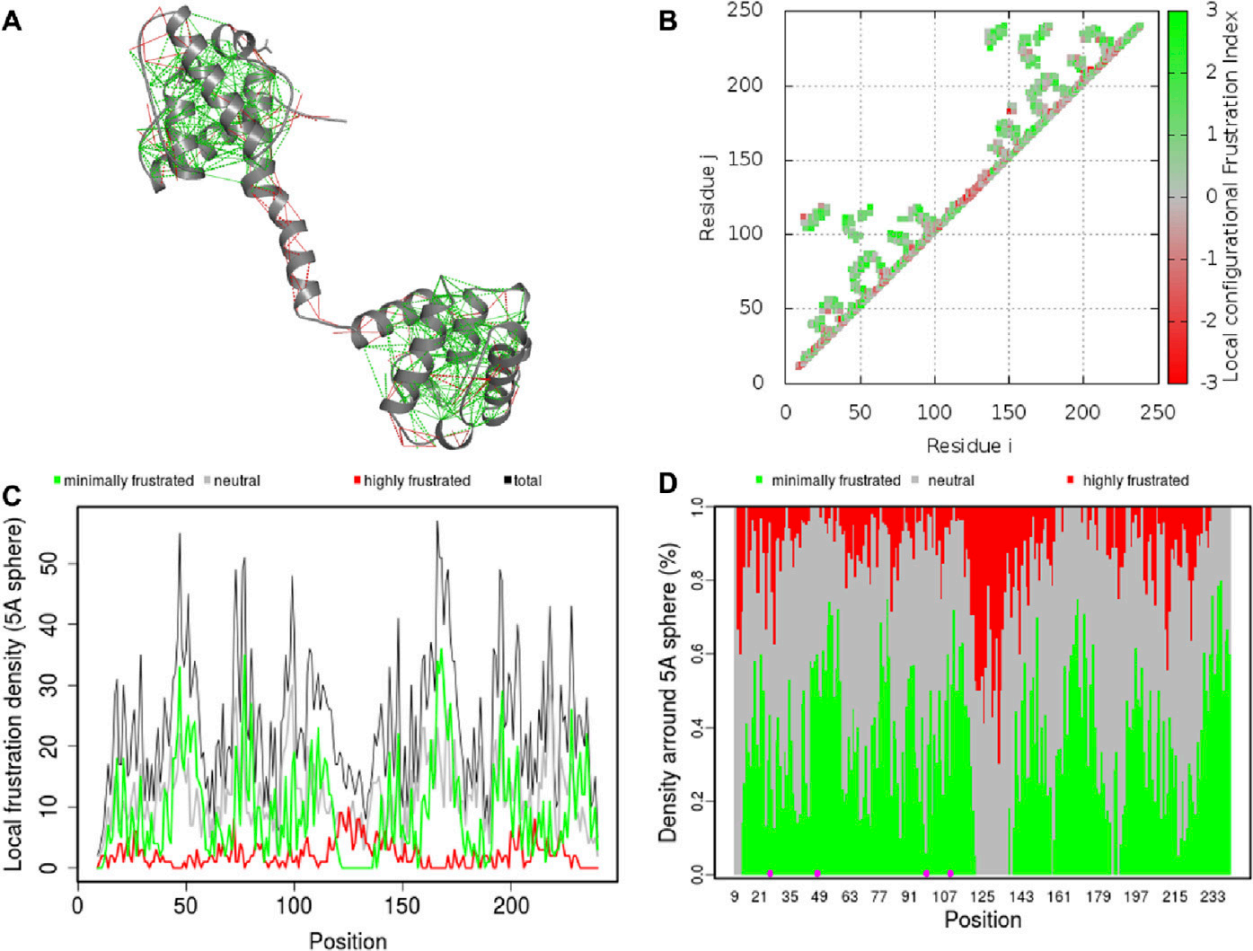
Residual frustration maps. **(A)** 3D structure of Dystrophin with frustration index. **(B)** Residue–residue contact level in Dystrophin. **(C)** The frustration contact map in Dystrophin. **(D)** The pointed frustration contact map of Dystrophin where mutation sites are highlighted in magenta.

Residual frustration analysis offers useful information about the topological features of energy landscapes of proteins and can be used to explain the connection between protein structure, stability, and function (Contessoto et al., 2013). Frustration analysis within protein structures provides information about locations of frustration. In this regard, we performed a comprehensive study of local frustration of the protein and identified frustration in Dystrophin (Figure 7). Frustration indices provide information about the relative stability of native contacts concerning all possible contacts at certain sites, which depend on the level of frustration. We observed that there are different levels of frustration in Dystrophin (Figure 7A). In addition, we analyzed configurational frustration at the residue-residue contact level in Dystrophin (Figure 7B). The contact map revealed that there was a general similarity in frustration patterns. The structure also exhibited moderate levels of frustration at many points (Figure 7C). Moreover, contacts involving mutated residues N26H, N26K, G47W, D98G, G109A, and G109R (highlighted within dashed circles in Figure 7D) demonstrated minimal frustration. These minimally frustrated residues of Dystrophin can be mutated and may affect the stability of the protein and, hence, the function of the protein causing DMD.

### Protein-protein interaction and functional characterization

**FIGURE 8:**
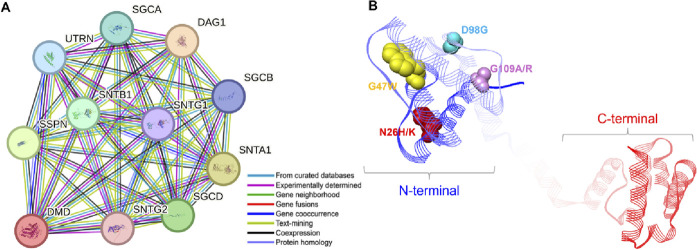
Protein-protein interaction and mutational landscape of Dystrophin. **(A)** Examination of the Dystrophin protein-protein interaction network with its associated partners. The network was constructed using STRING with a confidence level of 0.700. **(B)** Localization of mutations on the structure of Dystrophin where all the elucidated mutations are located on the N-terminal of the protein.

To examine the regulatory mechanisms of the abnormally expressed Dystrophin protein, it is important to study its associations with other proteins. Based on the STRING database of protein-protein interactions, we found several proteins that are closely connected (Figure 8). The study revealed that Dystrophin binds to SGCA, SGCB, and SGCD, thereby connecting it to the sarcoglycans, a family of proteins that plays a critical role in the structural integrity and function of muscle cells (Hack et al., 2000). These proteins are well known for their function as linkers of the muscle fiber cytoskeleton to the ECM and protecting the muscle fiber sarcolemma from shearing forces (Berthier and Blaineau, 1997). Furthermore, Dystrophin was shown to bind syntrophins SNTA1, SNTB1, SNTG1, and SNTG2 (Figure 8A), which are crucial for assembling and stabilizing the DGC in muscle cells (Bhat et al., 2013). These interactions are crucial for maintaining the structural and signaling roles of muscles (Bhat et al., 2019). Another interesting interaction emerged with DAG1, also known as dystroglycan, which is a critical component involved in maintaining the structural and functional integrity of muscle cells (Sciandra et al., 2007). Further, Dystrophin binds with UTRN (utrophin) and SSPN (sarcospan), both of which are crucial proteins involved in the structural and functional maintenance of muscle cells (Peter et al., 2008). It is evident from the above information that mutations within Dystrophin are directly associated with DMD (Muntoni et al., 2003). Our study pinpointed six specific mutations in the conserved region of Dystrophin: The amino acid substitutions include N26H, N26K, G47W, D98G, G109A, and G109R (Figure 8B). These mutations occur within the region of the protein that is highly conserved and is involved in actin binding at the N terminus of Dystrophin, which makes them pathogenic, destabilizing, and damaging. They can cause protein aggregation and are in the least frustrated protein domains, which might contribute to the Dystrophin-associated DMD pathogenesis.

## Conclusion

Dystrophin is involved in the structural integrity and function of muscle fibers and is known to interact with the cytoskeleton and the extracellular matrix. Mutations in the *Dystrophin* gene can be pathogenic, resulting in DMD, which is characterized by progressive muscle weakening and degeneration. This work offers a thorough analysis of the effects of pathogenic single-point mutations in Dystrophin on DMD development. To achieve this, we used a combination of sequence-based and structure-based computational tools and identified a set of high-confidence mutations that are likely to cause a severe disruption of the Dystrophin structure and function. Functional annotation tools, including CADD, allele frequency analysis, and PTM predictions, were also exploited to identify high-confidence deleterious mutations in Dystrophin. We have identified six substitutions (N26H, N26K, G47W, D98G, G109A, and G109R) that can decrease the solubility of the protein and are in the minimally frustrated conserved region of the protein, which may affect Dystrophin’s function and contribute to DMD development. The deleterious impact of mutations like N26H, N26K, G47W, D98G, G109A, and G109R in Dystrophin can lead to a breakdown in crucial protein-protein interactions, potentially exacerbating DMD development. The findings of the study are important for the current research aimed at identifying the disease mechanism and developing targeted treatment approaches for patients with this severe neuromuscular disorder. Future investigations can incorporate this *in silico* data to conduct a more thorough analysis of its biological significance in DMD pathogenesis.

## Data Availability

The original contributions presented in the study are included in the article/Supplementary Material, further inquiries can be directed to the corresponding authors.
