# Supplementary material for: A structural genomics approach to investigate Dystrophin mutations and their impact on the molecular pathways of Duchenne muscular dystrophy
Source: Front Genet. 2025 Feb 4;16:1517707. doi: 10.3389/fgene.2025.1517707 (PMC11841421; doi:10.3389/fgene.2025.1517707)
Supplement: Supplementary file 1 [file Table1.docx]

*Article*

**A Structural Genomics Approach to Investigate Dystrophin Mutations and their Impact on the Molecular Pathways of Duchenne Muscular Dystrophy**

Abdelbaset Mohamed Elasbali^1^, Farah Anjum^2^, Osama A AlKhamees^3^, Waleed Abu Al-Soud^4^, Mohd Adnan^5^, Anas Shamsi^6^ and Md. Imtaiyaz Hassan^7,*^

*^1^Department of Clinical Laboratory Science, College of Applied Medical Sciences-Qurayyat, Jouf University, Saudi Arabia.*

*^2^Department of Clinical Laboratory Sciences, College of Applied Medical Sciences, Taif University, PO Box 11099, 21944, Taif, Saudi Arabia.*

*^3^Department of Pharmacology, College of Medicine, Imam Mohammad Ibn Saud Islamic University (IMSIU), Riyadh 5701, Saudi Arabia.*

*^4^Molekylärbiologi, Klinisk Mikrobiologi och vårdhygien, Region Skåne, Sölvegatan 23B, 221 85 Lund, Sweden.*

*^5^Department of Biology, College of Science, University of Ha'il, Ha'il, Saudi Arabia.*

*^6^Centre of Medical and Bio-Allied Health Sciences Research, Ajman University, Ajman, United Arab Emirates.*

*^7^Centre for Interdisciplinary Research in Basic Sciences, Jamia Millia Islamia, New Delhi 110025, India.*

****Corresponding Author***

**Md. Imtaiyaz Hassan, Ph.D., FRSB., FRSC.**

Professor

Centre for Interdisciplinary Research in Basic Sciences

Jamia Millia Islamia, Jamia Nagar, New Delhi 110025, INDIA

E-mail: mihassan@jmi.ac.in

**Table S1**: Prediction of deleterious mutations in Dystrophin using sequence-based tools.

| **S. No.** | **Mutation** | **SIFT Class** | **PolyPhen Class** | **FATHMM** | **SNPs&GO** |
| --- | --- | --- | --- | --- | --- |
|  | A129T | Deleterious | Benign | Tolerated | Neutral |
|  | A168D | Deleterious | Probably Damaging | Damaging | Disease |
|  | A171P | Deleterious | Probably Damaging | Damaging | Disease |
|  | A171T | Deleterious | Probably Damaging | Damaging | Disease |
|  | A199E | Deleterious | Benign | Damaging | Disease |
|  | A199P | Deleterious | Possibly Damaging | Damaging | Disease |
|  | A203T | Deleterious | Possibly Damaging | Damaging | Disease |
|  | A27E | Deleterious | Benign | Tolerated | Disease |
|  | A27P | Deleterious | Benign | Tolerated | Disease |
|  | A27V | Deleterious | Benign | Tolerated | Disease |
|  | A80T | Deleterious | Benign | Damaging | Disease |
|  | C188G | Tolerated - Low Confidence | Benign | Tolerated | Neutral |
|  | C188W | Deleterious - Low Confidence | Benign | Tolerated | Disease |
|  | D101N | Deleterious | Possibly Damaging | Damaging | Disease |
|  | D15H | Deleterious | Possibly Damaging | Tolerated | Neutral |
|  | D165V | Deleterious | Possibly Damaging | Damaging | Disease |
|  | D165Y | Deleterious | Probably Damaging | Damaging | Disease |
|  | D179E | Tolerated | Benign | Tolerated | Neutral |
|  | D214N | Deleterious | Benign | Damaging | Disease |
|  | D219E | Deleterious | Benign | Tolerated | Neutral |
|  | D46N | Deleterious | Benign | Damaging | Disease |
|  | D46V | Deleterious | Benign | Damaging | Disease |
|  | D52Y | Deleterious | Benign | Tolerated | Disease |
|  | D98G | Deleterious | Probably Damaging | Damaging | Disease |
|  | E14K | Deleterious | Benign | Tolerated | Disease |
|  | E197K | Deleterious | Benign | Tolerated | Disease |
|  | E210D | Tolerated | Benign | Tolerated | Neutral |
|  | E38D | Tolerated | Benign | Tolerated | Neutral |
|  | E55K | Deleterious | Benign | Damaging | Disease |
|  | E65D | Deleterious | Benign | Tolerated | Neutral |
|  | F21S | Deleterious | Probably Damaging | Damaging | Disease |
|  | G102R | Deleterious | Probably Damaging | Damaging | Disease |
|  | G109A | Deleterious | Probably Damaging | Damaging | Disease |
|  | G109D | Deleterious | Probably Damaging | Damaging | Disease |
|  | G109R | Deleterious | Probably Damaging | Damaging | Disease |
|  | G130E | Deleterious | Benign | Tolerated | Neutral |
|  | G166V | Deleterious | Probably Damaging | Damaging | Disease |
|  | G47A | Deleterious | Benign | Damaging | Disease |
|  | G47E | Deleterious | Benign | Damaging | Disease |
|  | G47W | Deleterious | Probably Damaging | Damaging | Disease |
|  | G95V | Deleterious | Probably Damaging | Damaging | Disease |
|  | H104N | Deleterious | Probably Damaging | Tolerated | Disease |
|  | H117Q | Deleterious | Probably Damaging | Tolerated | Disease |
|  | H174R | Deleterious | Possibly Damaging | Damaging | Disease |
|  | H176N | Deleterious | Possibly Damaging | Damaging | Disease |
|  | H198R | Deleterious | Benign | Tolerated | Disease |
|  | H36L | Tolerated | Benign | Tolerated | Neutral |
|  | I111L | Deleterious | Possibly Damaging | Tolerated | Neutral |
|  | I111N | Deleterious | Probably Damaging | Damaging | Disease |
|  | I115V | Deleterious | Possibly Damaging | Damaging | Disease |
|  | I127V | Tolerated | Benign | Tolerated | Neutral |
|  | I157M | Deleterious | Possibly Damaging | Tolerated | Neutral |
|  | I157V | Tolerated | Benign | Tolerated | Neutral |
|  | I173V | Deleterious | Benign | Tolerated | Neutral |
|  | I209T | Deleterious | Benign | Damaging | Disease |
|  | I209V | Deleterious | Benign | Tolerated | Neutral |
|  | I228N | Deleterious | Probably Damaging | Tolerated | Disease |
|  | I228V | Tolerated | Benign | Tolerated | Neutral |
|  | I232L | Deleterious | Benign | Tolerated | Neutral |
|  | I232M | Deleterious | Benign | Damaging | Neutral |
|  | I37L | Deleterious | Benign | Tolerated | Disease |
|  | I37V | Tolerated | Benign | Tolerated | Neutral |
|  | I94T | Deleterious | Probably Damaging | Damaging | Disease |
|  | I99V | Deleterious | Possibly Damaging | Damaging | Disease |
|  | K105I | Deleterious | Probably Damaging | Damaging | Disease |
|  | K105N | Deleterious | Probably Damaging | Damaging | Disease |
|  | K121R | Deleterious | Possibly Damaging | Tolerated | Disease |
|  | K125I | Deleterious | Probably Damaging | Tolerated | Disease |
|  | K18N | Deleterious | Probably Damaging | Damaging | Disease |
|  | K34E | Deleterious | Benign | Tolerated | Disease |
|  | K64T | Deleterious | Possibly Damaging | Damaging | Neutral |
|  | K66T | Deleterious | Possibly Damaging | Damaging | Neutral |
|  | L108P | Deleterious | Probably Damaging | Damaging | Disease |
|  | L140R | Deleterious | Probably Damaging | Damaging | Disease |
|  | L140V | Deleterious | Probably Damaging | Damaging | Disease |
|  | L172V | Deleterious | Benign | Damaging | Neutral |
|  | L235I | Deleterious | Benign | Tolerated | Neutral |
|  | L239F | Deleterious | Benign | Tolerated | Neutral |
|  | L40V | Deleterious | Benign | Damaging | Disease |
|  | L50H | Deleterious | Probably Damaging | Damaging | Disease |
|  | L51P | Deleterious | Probably Damaging | Damaging | Disease |
|  | L54R | Deleterious | Benign | Damaging | Disease |
|  | L57P | Deleterious | Probably Damaging | Damaging | Disease |
|  | L57R | Deleterious | Benign | Damaging | Disease |
|  | L81Q | Deleterious | Probably Damaging | Damaging | Disease |
|  | M124T | Deleterious | Possibly Damaging | Tolerated | Disease |
|  | M124V | Deleterious | Possibly Damaging | Tolerated | Disease |
|  | M128I | Deleterious | Benign | Tolerated | Disease |
|  | N103D | Deleterious | Probably Damaging | Damaging | Disease |
|  | N113S | Tolerated | Benign | Tolerated | Neutral |
|  | N122T | Deleterious | Benign | Tolerated | Neutral |
|  | N126D | Tolerated | Benign | Tolerated | Neutral |
|  | N126S | Tolerated | Benign | Tolerated | Neutral |
|  | N135D | Deleterious | Possibly Damaging | Tolerated | Disease |
|  | N135S | Deleterious | Benign | Tolerated | Neutral |
|  | N150H | Tolerated | Benign | Tolerated | Neutral |
|  | N155D | Deleterious | Possibly Damaging | Tolerated | Disease |
|  | N158K | Deleterious | Possibly Damaging | Damaging | Disease |
|  | N184S | Tolerated | Benign | Tolerated | Neutral |
|  | N201I | Deleterious | Benign | Damaging | Disease |
|  | N201K | Deleterious | Benign | Tolerated | Neutral |
|  | N26D | Deleterious | Probably Damaging | Damaging | Disease |
|  | N26H | Deleterious | Probably Damaging | Damaging | Disease |
|  | N26K | Deleterious | Possibly Damaging | Damaging | Disease |
|  | N75K | Deleterious | Possibly Damaging | Tolerated | Disease |
|  | N75S | Deleterious | Possibly Damaging | Tolerated | Disease |
|  | N87S | Deleterious | Benign | Tolerated | Disease |
|  | N93D | Deleterious | Probably Damaging | Damaging | Disease |
|  | P223L | Deleterious | Benign | Damaging | Disease |
|  | P240L | Deleterious | Benign | Damaging | Disease |
|  | P240T | Deleterious | Benign | Damaging | Disease |
|  | Q132R | Deleterious | Possibly Damaging | Tolerated | Disease |
|  | Q133E | Deleterious | Possibly Damaging | Tolerated | Disease |
|  | Q133H | Deleterious | Probably Damaging | Damaging | Disease |
|  | Q133P | Deleterious | Probably Damaging | Damaging | Disease |
|  | Q153R | Deleterious | Benign | Tolerated | Disease |
|  | Q189H | Deleterious | Benign | Tolerated | Neutral |
|  | Q190R | Tolerated | Benign | Tolerated | Neutral |
|  | Q194R | Deleterious | Benign | Tolerated | Neutral |
|  | Q206L | Deleterious | Benign | Tolerated | Neutral |
|  | Q237H | Deleterious | Benign | Tolerated | Neutral |
|  | Q28E | Deleterious | Possibly Damaging | Tolerated | Disease |
|  | Q35P | Tolerated | Benign | Tolerated | Neutral |
|  | Q85E | Deleterious | Benign | Tolerated | Neutral |
|  | Q85R | Deleterious | Benign | Tolerated | Neutral |
|  | R13K | Deleterious | Probably Damaging | Tolerated | Neutral |
|  | R145L | Deleterious | Probably Damaging | Damaging | Disease |
|  | R145P | Deleterious | Probably Damaging | Damaging | Disease |
|  | R145Q | Deleterious | Possibly Damaging | Tolerated | Disease |
|  | R149C | Deleterious | Probably Damaging | Damaging | Disease |
|  | R149H | Deleterious | Possibly Damaging | Tolerated | Disease |
|  | R149S | Deleterious | Possibly Damaging | Tolerated | Disease |
|  | R204S | Deleterious | Benign | Tolerated | Neutral |
|  | R48G | Deleterious | Benign | Damaging | Disease |
|  | R48K | Deleterious | Benign | Tolerated | Neutral |
|  | R49C | Deleterious | Benign | Tolerated | Disease |
|  | R49H | Deleterious | Benign | Tolerated | Neutral |
|  | R82P | Deleterious | Benign | Tolerated | Disease |
|  | R82Q | Tolerated | Benign | Tolerated | Neutral |
|  | R82W | Deleterious | Benign | Tolerated | Neutral |
|  | S142C | Deleterious | Probably Damaging | Damaging | Disease |
|  | S142R | Deleterious | Possibly Damaging | Tolerated | Disease |
|  | S164C | Deleterious | Probably Damaging | Damaging | Disease |
|  | S175G | Deleterious | Possibly Damaging | Tolerated | Neutral |
|  | S175N | Deleterious | Benign | Tolerated | Disease |
|  | S185N | Tolerated | Benign | Tolerated | Neutral |
|  | S185T | Tolerated | Benign | Tolerated | Neutral |
|  | S227C | Deleterious | Possibly Damaging | Damaging | Disease |
|  | S227F | Deleterious | Benign | Damaging | Disease |
|  | S42N | Tolerated | Benign | Tolerated | Neutral |
|  | S96N | Deleterious | Benign | Tolerated | Neutral |
|  | T148I | Deleterious | Possibly Damaging | Tolerated | Disease |
|  | T148P | Deleterious | Probably Damaging | Damaging | Disease |
|  | T160I | Deleterious | Probably Damaging | Damaging | Disease |
|  | T160N | Deleterious | Probably Damaging | Damaging | Disease |
|  | T160P | Deleterious | Probably Damaging | Damaging | Disease |
|  | T161I | Deleterious | Benign | Damaging | Disease |
|  | T193A | Tolerated | Benign | Tolerated | Neutral |
|  | T193I | Tolerated | Benign | Tolerated | Neutral |
|  | T20A | Deleterious | Possibly Damaging | Tolerated | Disease |
|  | T220A | Deleterious | Benign | Tolerated | Neutral |
|  | T220N | Deleterious | Probably Damaging | Tolerated | Neutral |
|  | T22K | Deleterious | Possibly Damaging | Damaging | Disease |
|  | T233A | Deleterious | Benign | Tolerated | Disease |
|  | T233I | Deleterious | Possibly Damaging | Tolerated | Disease |
|  | T97A | Tolerated | Benign | Tolerated | Neutral |
|  | V100I | Deleterious | Probably Damaging | Tolerated | Disease |
|  | V123I | Tolerated | Benign | Tolerated | Neutral |
|  | V186M | Deleterious | Benign | Damaging | Neutral |
|  | V187I | Tolerated | Benign | Tolerated | Neutral |
|  | V25A | Deleterious | Benign | Tolerated | Neutral |
|  | V77D | Deleterious | Possibly Damaging | Damaging | Disease |
|  | V83F | Tolerated | Benign | Tolerated | Neutral |
|  | V83I | Tolerated | Benign | Tolerated | Neutral |
|  | V92A | Deleterious | Possibly Damaging | Damaging | Disease |
|  | V92M | Deleterious | Benign | Damaging | Disease |
|  | W118R | Deleterious | Probably Damaging | Tolerated | Disease |
|  | W143C | Deleterious | Probably Damaging | Damaging | Disease |
|  | W143L | Deleterious | Probably Damaging | Damaging | Disease |
|  | W163R | Deleterious | Probably Damaging | Damaging | Disease |
|  | W183R | Deleterious | Probably Damaging | Tolerated | Disease |
|  | Y205H | Tolerated | Benign | Tolerated | Neutral |
|  | Y231C | Deleterious | Benign | Damaging | Disease |
|  | Y231N | Deleterious | Possibly Damaging | Damaging | Disease |

**Table S2**: Prediction of destabilizing mutations in Dystrophin using structure-based tools.

| **S. No** | **Mutation** | **mCSM** | **DynaMut2** | **MAESTROweb** | **PremPS** |
| --- | --- | --- | --- | --- | --- |
|  | A129T | Destabilizing | −1.07 | 0.844 | 0.24 |
|  | A168D | Highly Destabilizing | −1.09 | 0.832 | 1.05 |
|  | A171P | Destabilizing | −0.21 | 0.85 | 0.88 |
|  | A171T | Destabilizing | −1.46 | 0.841 | 0.69 |
|  | A199E | Highly Destabilizing | −0.92 | 0.828 | 1.4 |
|  | A199P | Destabilizing | −0.2 | 0.828 | 0.93 |
|  | A203T | Destabilizing | −1.55 | 0.839 | 0.86 |
|  | A27E | Destabilizing | −1 | 0.828 | 0.71 |
|  | A27P | Destabilizing | −0.59 | 0.857 | 0.68 |
|  | A27V | Destabilizing | −1.15 | 0.881 | 0.43 |
|  | A80T | Destabilizing | −1.55 | 0.843 | 0.47 |
|  | C188G | Destabilizing | −0.98 | 0.952 | 0.39 |
|  | C188W | Destabilizing | −0.9 | 0.894 | 0.26 |
|  | D101N | Stabilizing | −0.03 | 0.888 | 0.1 |
|  | D15H | Destabilizing | −0.47 | 0.895 | -0.07 |
|  | D165V | Destabilizing | −1.06 | 0.892 | -0.56 |
|  | D165Y | Destabilizing | −1.07 | 0.896 | -0.2 |
|  | D179E | Destabilizing | −0.22 | 0.891 | 0.11 |
|  | D214N | Destabilizing | −0.64 | 0.883 | 0.23 |
|  | D219E | Destabilizing | −0.94 | 0.883 | 0.17 |
|  | D46N | Destabilizing | −1.69 | 0.894 | 0.27 |
|  | D46V | Stabilizing | 0.76 | 0.841 | -0.5 |
|  | D52Y | Stabilizing | 0.26 | 0.825 | 0.06 |
|  | D98G | Destabilizing | −0.48 | 0.88 | 0.35 |
|  | E14K | Destabilizing | −0.67 | 0.834 | 0.18 |
|  | E197K | Destabilizing | −0.67 | 0.838 | 0.24 |
|  | E210D | Destabilizing | −0.37 | 0.881 | 0.34 |
|  | E38D | Destabilizing | −0.1 | 0.863 | 0.1 |
|  | E55K | Destabilizing | −0.63 | 0.803 | 0.62 |
|  | E65D | Destabilizing | −1.16 | 0.918 | 0.67 |
|  | F21S | Highly Destabilizing | −2.47 | 0.796 | 2.54 |
|  | G102R | Destabilizing | −1.02 | 0.863 | 0.3 |
|  | G109A | Destabilizing | −0.57 | 0.903 | 0.48 |
|  | G109D | Destabilizing | −1.42 | 0.831 | 0.29 |
|  | G109R | Destabilizing | −1.28 | 0.917 | 0.37 |
|  | G130E | Destabilizing | −0.23 | 0.846 | -0.2 |
|  | G166V | Destabilizing | −1.92 | 0.853 | -0.11 |
|  | G47A | Destabilizing | −1.02 | 0.883 | 0.79 |
|  | G47E | Highly Destabilizing | −0.94 | 0.824 | 1.06 |
|  | G47W | Destabilizing | −0.88 | 0.97 | 0.4 |
|  | G95V | Destabilizing | −0.67 | 0.854 | 0.52 |
|  | H104N | Destabilizing | −0.16 | 0.819 | 0.69 |
|  | H117Q | Destabilizing | −0.9 | 0.841 | 0.16 |
|  | H174R | Destabilizing | −0.07 | 0.867 | 0.82 |
|  | H176N | Destabilizing | −1.51 | 0.857 | 0.7 |
|  | H198R | Destabilizing | −1.06 | 0.898 | 0.42 |
|  | H36L | Stabilizing | 1.29 | 0.901 | 0.02 |
|  | I111L | Destabilizing | −0.27 | 0.881 | 0.69 |
|  | I111N | Highly Destabilizing | −2.34 | 0.82 | 2.58 |
|  | I115V | Destabilizing | −1.17 | 0.85 | 0.78 |
|  | I127V | Destabilizing | −0.98 | 0.934 | 0.32 |
|  | I157M | Destabilizing | −0.64 | 0.869 | 0.18 |
|  | I157V | Destabilizing | −0.31 | 0.9 | 0.3 |
|  | I173V | Destabilizing | −1.14 | 0.844 | 1.01 |
|  | I209T | Highly Destabilizing | −1.99 | 0.813 | 2.02 |
|  | I209V | Destabilizing | −1.12 | 0.868 | 0.57 |
|  | I228N | Highly Destabilizing | −2.32 | 0.849 | 2.67 |
|  | I228V | Destabilizing | −1.29 | 0.844 | 0.61 |
|  | I232L | Destabilizing | −0.25 | 0.888 | 0.67 |
|  | I232M | Destabilizing | −0.7 | 0.843 | 1.06 |
|  | I37L | Destabilizing | −0.76 | 0.911 | 0.78 |
|  | I37V | Destabilizing | −1.42 | 0.851 | 0.38 |
|  | I94T | Destabilizing | −2.04 | 0.825 | 1.98 |
|  | I99V | Highly Destabilizing | −1.02 | 0.838 | 0.94 |
|  | K105I | Stabilizing | 1 | 0.902 | -0.22 |
|  | K105N | Destabilizing | 0.18 | 0.921 | 0.24 |
|  | K121R | Destabilizing | −0.83 | 0.878 | -0.12 |
|  | K125I | Stabilizing | 1 | 0.884 | -0.28 |
|  | K18N | Destabilizing | −1.6 | 0.808 | 0.85 |
|  | K34E | Stabilizing | −0.15 | 0.842 | 0.89 |
|  | K64T | Destabilizing | −0.63 | 0.842 | 0.62 |
|  | K66T | Stabilizing | −0.09 | 0.854 | 0.03 |
|  | L108P | Destabilizing | −0.5 | 0.775 | 2.42 |
|  | L140R | Destabilizing | −1.5 | 0.815 | 2.31 |
|  | L140V | Destabilizing | −0.88 | 0.843 | 0.93 |
|  | L172V | Destabilizing | −1.41 | 0.847 | 0.5 |
|  | L235I | Destabilizing | −0.63 | 0.837 | 0.83 |
|  | L239F | Destabilizing | −0.99 | 0.9 | 0.3 |
|  | L40V | Destabilizing | −1.39 | 0.846 | 1.05 |
|  | L50H | Highly Destabilizing | −0.61 | 0.79 | 2.55 |
|  | L51P | Highly Destabilizing | −1.23 | 0.802 | 2.97 |
|  | L54R | Highly Destabilizing | −1.34 | 0.804 | 2.59 |
|  | L57P | Destabilizing | −0.53 | 0.859 | 2 |
|  | L57R | Destabilizing | −1.26 | 0.817 | 1.78 |
|  | L81Q | Highly Destabilizing | −1.86 | 0.871 | 1.75 |
|  | M124T | Destabilizing | 0.07 | 0.92 | 0.35 |
|  | M124V | Destabilizing | −0.49 | 0.916 | 0.3 |
|  | M128I | Destabilizing | −0.65 | 0.909 | 0.37 |
|  | N103D | Destabilizing | −1 | 0.872 | 0.65 |
|  | N113S | Destabilizing | −1.3 | 0.871 | -0.37 |
|  | N122T | Destabilizing | −0.35 | 0.935 | 0.35 |
|  | N126D | Destabilizing | −0.21 | 0.874 | -0.17 |
|  | N126S | Destabilizing | 0 | 0.925 | 0.05 |
|  | N135D | Stabilizing | 0.14 | 0.872 | 0.73 |
|  | N135S | Destabilizing | 0.24 | 0.884 | 0.42 |
|  | N150H | Destabilizing | −0.61 | 0.899 | 0.1 |
|  | N155D | Stabilizing | 0.27 | 0.831 | 0.18 |
|  | N158K | Stabilizing | −0.05 | 0.867 | 0.51 |
|  | N184S | Destabilizing | 0.04 | 0.922 | 0.16 |
|  | N201I | Stabilizing | 0.29 | 0.82 | 0.2 |
|  | N201K | Destabilizing | −0.71 | 0.9 | 0.48 |
|  | N26D | Destabilizing | −0.81 | 0.842 | 0.96 |
|  | N26H | Destabilizing | −1.57 | 0.849 | 0.73 |
|  | N26K | Destabilizing | −1 | 0.888 | 0.59 |
|  | N75K | Destabilizing | −0.65 | 0.903 | 0.59 |
|  | N75S | Destabilizing | −0.45 | 0.853 | 0.51 |
|  | N87S | Destabilizing | −1.41 | 0.865 | 0.66 |
|  | N93D | Stabilizing | 0.19 | 0.811 | 0.17 |
|  | P223L | Destabilizing | −1.39 | 0.956 | 0.05 |
|  | P240L | Destabilizing | −0.88 | 0.857 | -0.22 |
|  | P240T | Destabilizing | −1.05 | 0.855 | 0 |
|  | Q132R | Stabilizing | 0.02 | 0.857 | 0.07 |
|  | Q133E | Destabilizing | 0.04 | 0.892 | 0.16 |
|  | Q133H | Destabilizing | −0.43 | 0.871 | 0.07 |
|  | Q133P | Stabilizing | −0.15 | 0.924 | 0.11 |
|  | Q153R | Destabilizing | −0.33 | 0.874 | 0.2 |
|  | Q189H | Destabilizing | −1.5 | 0.884 | 0.29 |
|  | Q190R | Stabilizing | 0.18 | 0.884 | -0.04 |
|  | Q194R | Destabilizing | −0.78 | 0.849 | 0.32 |
|  | Q206L | Stabilizing | 0.26 | 0.902 | 0 |
|  | Q237H | Destabilizing | −0.55 | 0.9 | 0.02 |
|  | Q28E | Destabilizing | −0.65 | 0.834 | 0.86 |
|  | Q35P | Stabilizing | −0.21 | 0.856 | -0.31 |
|  | Q85E | Destabilizing | −0.83 | 0.91 | 0.03 |
|  | Q85R | Destabilizing | −0.89 | 0.906 | -0.04 |
|  | R13K | Destabilizing | −0.21 | 0.809 | 0.4 |
|  | R145L | Destabilizing | −0.67 | 0.886 | 0.21 |
|  | R145P | Destabilizing | −0.51 | 0.858 | 0.37 |
|  | R145Q | Destabilizing | −1.44 | 0.892 | 0.3 |
|  | R149C | Destabilizing | −0.08 | 0.854 | 0.13 |
|  | R149H | Destabilizing | −1.93 | 0.862 | 0.28 |
|  | R149S | Destabilizing | −0.38 | 0.882 | 0.05 |
|  | R204S | Destabilizing | −2.14 | 0.841 | 0.83 |
|  | R48G | Destabilizing | −1.33 | 0.822 | 0.83 |
|  | R48K | Destabilizing | −1.81 | 0.803 | 0.47 |
|  | R49C | Destabilizing | −1.44 | 0.84 | 0.95 |
|  | R49H | Highly Destabilizing | −0.55 | 0.817 | 1.17 |
|  | R82P | Stabilizing | 0.21 | 0.92 | 0.11 |
|  | R82Q | Destabilizing | −0.16 | 0.858 | -0.23 |
|  | R82W | Destabilizing | −0.66 | 0.849 | 0.11 |
|  | S142C | Destabilizing | −0.7 | 0.934 | 0.43 |
|  | S142R | Destabilizing | −0.85 | 0.925 | 0.5 |
|  | S164C | Destabilizing | −1.03 | 0.841 | 0.2 |
|  | S175G | Destabilizing | −1.24 | 0.889 | 0.59 |
|  | S175N | Destabilizing | −1.31 | 0.859 | 0.39 |
|  | S185N | Destabilizing | −1.15 | 0.867 | 0.14 |
|  | S185T | Destabilizing | −0.6 | 0.946 | 0.01 |
|  | S227C | Destabilizing | −0.68 | 0.842 | 0.55 |
|  | S227F | Destabilizing | −0.88 | 0.874 | -0.15 |
|  | S42N | Destabilizing | −0.08 | 0.886 | 0.36 |
|  | S96N | Destabilizing | −1.12 | 0.865 | 0.53 |
|  | T148I | Destabilizing | −0.74 | 0.853 | 0.44 |
|  | T148P | Destabilizing | −0.81 | 0.835 | 1.18 |
|  | T160I | Destabilizing | −0.6 | 0.942 | 0.31 |
|  | T160N | Destabilizing | −1.49 | 0.861 | 0.77 |
|  | T160P | Destabilizing | −0.39 | 0.856 | 0.62 |
|  | T161I | Destabilizing | −0.94 | 0.875 | 0.32 |
|  | T193A | Destabilizing | −1.19 | 0.866 | 0.47 |
|  | T193I | Destabilizing | −0.74 | 0.854 | -0.45 |
|  | T20A | Destabilizing | −1.27 | 0.926 | 0.58 |
|  | T220A | Destabilizing | −0.94 | 0.856 | 0.55 |
|  | T220N | Destabilizing | −0.74 | 0.881 | 0.82 |
|  | T22K | Destabilizing | −0.72 | 0.913 | 1.06 |
|  | T233A | Destabilizing | −1.2 | 0.914 | 0.3 |
|  | T233I | Stabilizing | 0.22 | 0.864 | 0.18 |
|  | T97A | Destabilizing | −1.09 | 0.864 | 0.48 |
|  | V100I | Destabilizing | −0.79 | 0.88 | 0.15 |
|  | V123I | Destabilizing | −0.18 | 0.911 | -0.11 |
|  | V186M | Destabilizing | −0.56 | 0.89 | 0.68 |
|  | V187I | Destabilizing | −0.64 | 0.888 | 0.12 |
|  | V25A | Destabilizing | −1.39 | 0.848 | 2.05 |
|  | V77D | Highly Destabilizing | −2.69 | 0.831 | 3.17 |
|  | V83F | Destabilizing | −0.5 | 0.809 | 0.46 |
|  | V83I | Destabilizing | −0.83 | 0.892 | 0.11 |
|  | V92A | Destabilizing | −0.66 | 0.891 | 0.87 |
|  | V92M | Destabilizing | −0.62 | 0.873 | 0.37 |
|  | W118R | Highly Destabilizing | −1.96 | 0.775 | 0.14 |
|  | W143C | Destabilizing | −1.26 | 0.843 | 1.03 |
|  | W143L | Destabilizing | −1.53 | 0.849 | 0.4 |
|  | W163R | Highly Destabilizing | −1.95 | 0.807 | 1.85 |
|  | W183R | Highly Destabilizing | −2.15 | 0.797 | 1.77 |
|  | Y205H | Destabilizing | −0.17 | 0.868 | 0.1 |
|  | Y231C | Destabilizing | −0.44 | 0.84 | 2.61 |
|  | Y231N | Highly Destabilizing | −2.26 | 0.835 | 2.57 |

**Table S3**: Prediction of pathogenic mutations in Dystrophin using structure-based tools.

| **S. No.** | **Mutation** | **CADD score** | **PhD-SNP** | **MutPred2** | **MutPred2 remarks** |
| --- | --- | --- | --- | --- | --- |
|  | K18N | 29 | Disease | 0.596 | Loss of Helix (Pr = 0.29 \| P = 9.8e-03); Altered DNA binding (Pr = 0.25 \| P = 6.1e-03); Altered Transmembrane protein (Pr = 0.16 \| P = 0.01); Gain of N-linked glycosylation at K18 (Pr = 0.06 \| P = 0.02) |
|  | F21S | 29 | Disease | 0.9 | Gain of Intrinsic disorder (Pr = 0.32 \| P = 0.03); Altered DNA binding (Pr = 0.29 \| P = 3.6e-03); Altered Ordered interface (Pr = 0.26 \| P = 0.02); Altered Disordered interface (Pr = 0.26 \| P = 0.05); Loss of Allosteric site at W24 (Pr = 0.19 \| P = 0.05); Loss of Acetylation at K23 (Pr = 0.19 \| P = 0.05); Altered Transmembrane protein (Pr = 0.14 \| P = 0.02); Altered Stability (Pr = 0.13 \| P = 0.03); Gain of GPI-anchor amidation at N26 (Pr = 0.02 \| P = 0.02) |
|  | T22K | 23 | Disease | 0.695 | Gain of Helix (Pr = 0.30 \| P = 9.2e-03); Altered DNA binding (Pr = 0.27 \| P = 4.1e-03); Altered Ordered interface (Pr = 0.25 \| P = 0.02); Altered Disordered interface (Pr = 0.20 \| P = 0.03); Gain of Allosteric site at W24 (Pr = 0.19 \| P = 0.04); Altered Transmembrane protein (Pr = 0.14 \| P = 0.02); Gain of GPI-anchor amidation at N26 (Pr = 0.01 \| P = 0.03) |
|  | N26D | 22 | Disease | 0.802 | Loss of Acetylation at K31 (Pr = 0.27 \| P = 7.1e-03); Altered DNA binding (Pr = 0.26 \| P = 7.3e-03); Altered Ordered interface (Pr = 0.25 \| P = 0.03); Loss of GPI-anchor amidation at N26 (Pr = 0.01 \| P = 0.03) |
|  | N26H | 25 | Disease | 0.681 | Altered DNA binding (Pr = 0.27 \| P = 6.5e-03); Loss of Acetylation at K31 (Pr = 0.27 \| P = 8.1e-03); Altered Ordered interface (Pr = 0.24 \| P = 0.03); Loss of GPI-anchor amidation at N26 (Pr = 0.01 \| P = 0.03) |
|  | N26K | 23 | Disease | 0.708 | Gain of Acetylation at K31 (Pr = 0.30 \| P = 3.5e-03); Altered DNA binding (Pr = 0.29 \| P = 3.8e-03); Altered Disordered interface (Pr = 0.28 \| P = 0.04); Gain of Helix (Pr = 0.28 \| P = 0.02); Altered Ordered interface (Pr = 0.25 \| P = 0.02); Loss of GPI-anchor amidation at N26 (Pr = 0.01 \| P = 0.03) |
|  | G47W | 29 | Disease | 0.899 | Altered Coiled coil (Pr = 0.44 \| P = 5.8e-03); Altered Disordered interface (Pr = 0.28 \| P = 0.04); Gain of Allosteric site at G47 (Pr = 0.28 \| P = 4.2e-03) |
|  | L50H | 22 | Disease | 0.81 | Altered Stability (Pr = 0.19 \| P = 0.01); Altered Coiled coil (Pr = 0.12 \| P = 0.04) |
|  | L51P | 27 | Disease | 0.867 | Loss of Helix (Pr = 0.30 \| P = 5.3e-03); Altered Stability (Pr = 0.19 \| P = 0.01); Altered Coiled coil (Pr = 0.13 \| P = 0.04) |
|  | L57P | 28 | Disease | 0.891 | Gain of Intrinsic disorder (Pr = 0.36 \| P = 0.02); Altered Stability (Pr = 0.13 \| P = 0.03); Altered Coiled coil (Pr = 0.10 \| P = 0.04) |
|  | V77D | 22 | Disease | 0.823 | Gain of B-factor (Pr = 0.26 \| P = 0.02); Altered Stability (Pr = 0.16 \| P = 0.02); Altered Metal binding (Pr = 0.05 \| P = 0.05) |
|  | L81Q | 27 | Disease | 0.775 | Altered Transmembrane protein (Pr = 0.23 \| P = 2.7e-03); Altered Stability (Pr = 0.20 \| P = 0.01) |
|  | V92A | 24 | Disease | 0.375 | - |
|  | I94T | 26 | Disease | 0.825 | Altered Stability (Pr = 0.38 \| P = 3.4e-03); Altered Transmembrane protein (Pr = 0.28 \| P = 4.9e-04); Gain of Allosteric site at N93 (Pr = 0.19 \| P = 0.05) |
|  | G95V | 31 | Disease | 0.721 | Altered Transmembrane protein (Pr = 0.31 \| P = 8.3e-05); Loss of Strand (Pr = 0.27 \| P = 0.01) |
|  | D98G | 27 | Disease | 0.853 | Altered Transmembrane protein (Pr = 0.32 \| P = 6.8e-05); Altered Metal binding (Pr = 0.18 \| P = 0.03) |
|  | I99V | 24 | Disease | 0.329 | - |
|  | G102R | 31 | Disease | 0.908 | Altered Transmembrane protein (Pr = 0.27 \| P = 6.9e-04); Altered Ordered interface (Pr = 0.25 \| P = 0.03); Altered Metal binding (Pr = 0.19 \| P = 0.02) |
|  | N103D | 26 | Disease | 0.664 | Altered Metal binding (Pr = 0.34 \| P = 1.3e-03); Altered Transmembrane protein (Pr = 0.27 \| P = 6.3e-04) |
|  | L108P | 28 | Disease | 0.956 | Altered Ordered interface (Pr = 0.33 \| P = 6.1e-03); Loss of Helix (Pr = 0.32 \| P = 2.8e-03); Altered Transmembrane protein (Pr = 0.29 \| P = 3.6e-04); Altered Metal binding (Pr = 0.17 \| P = 0.03) |
|  | G109A | 26 | Disease | 0.843 | Gain of Helix (Pr = 0.31 \| P = 4.3e-03); Altered Transmembrane protein (Pr = 0.27 \| P = 5.0e-04); Altered Ordered interface (Pr = 0.26 \| P = 0.02) |
|  | G109D | 28 | Disease | 0.909 | Gain of Helix (Pr = 0.35 \| P = 7.2e-04); Altered Transmembrane protein (Pr = 0.28 \| P = 6.5e-04); Altered Ordered interface (Pr = 0.28 \| P = 0.03) |
|  | G109R | 28 | Disease | 0.883 | Altered Transmembrane protein (Pr = 0.33 \| P = 5.4e-05); Gain of Helix (Pr = 0.30 \| P = 9.6e-03); Altered Ordered interface (Pr = 0.27 \| P = 0.01); Altered Disordered interface (Pr = 0.19 \| P = 0.03) |
|  | I111N | 28 | Disease | 0.893 | Altered Transmembrane protein (Pr = 0.43 \| P = 0.0e+00); Altered Ordered interface (Pr = 0.27 \| P = 8.4e-03); Gain of Allosteric site at W112 (Pr = 0.22 \| P = 0.03) |
|  | I115V | 25 | Disease | 0.333 | - |
|  | Q133H | 24 | Benign | 0.314 | - |
|  | L140R | 29 | Disease | 0.862 | Altered Ordered interface (Pr = 0.25 \| P = 0.03); Altered Transmembrane protein (Pr = 0.21 \| P = 4.5e-03); Altered Disordered interface (Pr = 0.18 \| P = 0.04); Loss of GPI-anchor amidation at N135 (Pr = 0.02 \| P = 0.02) |
|  | L140V | 26 | Disease | 0.52 | Altered Transmembrane protein (Pr = 0.17 \| P = 0.01); Gain of GPI-anchor amidation at N135 (Pr = 0.02 \| P = 0.02) |
|  | S142C | 26 | Benign | 0.347 | - |
|  | W143C | 31 | Disease | 0.908 | Altered Ordered interface (Pr = 0.28 \| P = 0.04); Altered Transmembrane protein (Pr = 0.17 \| P = 9.3e-03); Altered DNA binding (Pr = 0.14 \| P = 0.05); Loss of Pyrrolidone carboxylic acid at Q146 (Pr = 0.05 \| P = 0.04) |
|  | W143L | 30 | Disease | 0.857 | Altered Ordered interface (Pr = 0.26 \| P = 0.01); Altered Transmembrane protein (Pr = 0.17 \| P = 9.5e-03); Altered DNA binding (Pr = 0.16 \| P = 0.04); Loss of Pyrrolidone carboxylic acid at Q146 (Pr = 0.05 \| P = 0.04) |
|  | R145L | 29 | Disease | 0.509 | Altered Ordered interface (Pr = 0.33 \| P = 9.2e-03); Altered Transmembrane protein (Pr = 0.29 \| P = 3.1e-04); Gain of ADP-ribosylation at R149 (Pr = 0.20 \| P = 0.04); Altered Metal binding (Pr = 0.11 \| P = 0.04); Loss of Pyrrolidone carboxylic acid at Q146 (Pr = 0.05 \| P = 0.04) |
|  | R145P | 32 | Disease | 0.821 | Altered Ordered interface (Pr = 0.32 \| P = 0.01); Altered Transmembrane protein (Pr = 0.28 \| P = 6.5e-04); Gain of ADP-ribosylation at R149 (Pr = 0.20 \| P = 0.04); Altered DNA binding (Pr = 0.16 \| P = 0.04); Loss of Pyrrolidone carboxylic acid at Q146 (Pr = 0.05 \| P = 0.04) |
|  | T148P | 27 | Disease | 0.816 | Altered Ordered interface (Pr = 0.28 \| P = 0.04); Altered Transmembrane protein (Pr = 0.25 \| P = 1.1e-03); Gain of ADP-ribosylation at R149 (Pr = 0.19 \| P = 0.04); Altered Metal binding (Pr = 0.10 \| P = 0.05); Loss of Pyrrolidone carboxylic acid at Q146 (Pr = 0.05 \| P = 0.04) |
|  | R149C | 33 | Disease | 0.478 | - |
|  | T160I | 27 | Disease | 0.795 | Altered Ordered interface (Pr = 0.29 \| P = 0.03); Gain of Allosteric site at W163 (Pr = 0.26 \| P = 9.1e-03); Altered Metal binding (Pr = 0.24 \| P = 0.02); Altered Transmembrane protein (Pr = 0.24 \| P = 1.5e-03); Altered DNA binding (Pr = 0.22 \| P = 0.01); Loss of Catalytic site at W163 (Pr = 0.20 \| P = 0.01); Loss of N-linked glycosylation at N158 (Pr = 0.07 \| P = 0.02) |
|  | T160N | 26 | Disease | 0.755 | Altered Ordered interface (Pr = 0.31 \| P = 0.01); Altered Transmembrane protein (Pr = 0.27 \| P = 8.7e-04); Gain of Relative solvent accessibility (Pr = 0.27 \| P = 0.02); Gain of Allosteric site at W163 (Pr = 0.25 \| P = 9.6e-03); Altered Metal binding (Pr = 0.24 \| P = 0.02); Altered DNA binding (Pr = 0.23 \| P = 0.01); Loss of Catalytic site at W163 (Pr = 0.21 \| P = 0.01); Loss of N-linked glycosylation at N158 (Pr = 0.07 \| P = 0.02) |
|  | T160P | 27 | Disease | 0.878 | Gain of Allosteric site at W163 (Pr = 0.31 \| P = 1.1e-03); Altered Ordered interface (Pr = 0.29 \| P = 0.03); Altered Metal binding (Pr = 0.24 \| P = 0.02); Altered Transmembrane protein (Pr = 0.24 \| P = 2.2e-03); Altered DNA binding (Pr = 0.23 \| P = 0.01); Loss of Catalytic site at W163 (Pr = 0.22 \| P = 9.4e-03); Loss of N-linked glycosylation at N158 (Pr = 0.07 \| P = 0.02) |
|  | T161I | 23 | Disease | 0.679 | Altered Ordered interface (Pr = 0.28 \| P = 0.04); Altered DNA binding (Pr = 0.26 \| P = 5.1e-03); Gain of Allosteric site at W163 (Pr = 0.26 \| P = 9.2e-03); Altered Transmembrane protein (Pr = 0.25 \| P = 1.3e-03); Altered Metal binding (Pr = 0.23 \| P = 0.02); Loss of Catalytic site at W163 (Pr = 0.19 \| P = 0.01); Gain of N-linked glycosylation at N158 (Pr = 0.06 \| P = 0.02) |
|  | W163R | 28 | Disease | 0.937 | Altered Ordered interface (Pr = 0.29 \| P = 0.03); Loss of Allosteric site at W163 (Pr = 0.29 \| P = 5.9e-03); Gain of Helix (Pr = 0.28 \| P = 0.03); Altered Metal binding (Pr = 0.25 \| P = 0.01); Gain of Catalytic site at W163 (Pr = 0.23 \| P = 7.5e-03); Altered DNA binding (Pr = 0.22 \| P = 0.01); Altered Transmembrane protein (Pr = 0.19 \| P = 7.5e-03); Loss of N-linked glycosylation at N158 (Pr = 0.06 \| P = 0.02) |
|  | S164C | 28 | Disease | 0.497 | - |
|  | A168D | 29 | Disease | 0.889 | Altered Ordered interface (Pr = 0.32 \| P = 9.6e-03); Altered Metal binding (Pr = 0.29 \| P = 3.4e-03); Altered DNA binding (Pr = 0.26 \| P = 5.5e-03); Gain of Allosteric site at W163 (Pr = 0.24 \| P = 0.01); Loss of Catalytic site at W163 (Pr = 0.20 \| P = 0.01); Altered Transmembrane protein (Pr = 0.16 \| P = 0.01) |
|  | A171P | 26 | Disease | 0.903 | Altered Metal binding (Pr = 0.27 \| P = 0.03); Gain of Allosteric site at H174 (Pr = 0.23 \| P = 0.02); Altered Transmembrane protein (Pr = 0.17 \| P = 9.4e-03) |
|  | A171T | 26 | Disease | 0.749 | Altered Metal binding (Pr = 0.30 \| P = 0.01); Altered Transmembrane protein (Pr = 0.17 \| P = 1.0e-02) |
|  | H174R | 25 | Disease | 0.889 | Altered Metal binding (Pr = 0.53 \| P = 3.3e-03); Gain of Allosteric site at H174 (Pr = 0.22 \| P = 0.03); Altered Transmembrane protein (Pr = 0.18 \| P = 8.8e-03) |
|  | H176N | 24 | Disease | 0.76 | Altered Metal binding (Pr = 0.35 \| P = 9.0e-03); Altered Transmembrane protein (Pr = 0.21 \| P = 4.2e-03); Gain of Allosteric site at H174 (Pr = 0.20 \| P = 0.04) |
|  | A199P | 27 | Disease | 0.896 | Loss of Helix (Pr = 0.29 \| P = 0.01) |
|  | A203T | 25 | Disease | 0.703 | Altered Transmembrane protein (Pr = 0.10 \| P = 0.05); Gain of N-linked glycosylation at N201 (Pr = 0.06 \| P = 0.02) |
|  | S227C | 26 | Disease | 0.623 | Altered Ordered interface (Pr = 0.24 \| P = 0.05); Loss of Sulfation at Y222 (Pr = 0.03 \| P = 0.01) |
|  | Y231N | 26 | Disease | 0.784 | Altered Ordered interface (Pr = 0.30 \| P = 4.7e-03); Altered Transmembrane protein (Pr = 0.16 \| P = 0.01); Gain of N-linked glycosylation at Y231 (Pr = 0.02 \| P = 0.03) |

**Table S4**: Allele frequency of the selected mutations retrieved from gnomAD database. Among the 50 mutations screened, allele frequency data were available for only 15 mutations in the gnomAD database.

| **S. No.** | **Mutation** | **Allele Frequency** |
| --- | --- | --- |
|  | F21S | 8.39E-07 |
|  | G95V | 9.97621E-06 |
|  | D98G | 2.48649E-06 |
|  | I99V | 0.000232038 |
|  | G102R | 8.29E-07 |
|  | G109A | 1.9045E-05 |
|  | Q133H | 8.26E-07 |
|  | L140V | 3.30655E-06 |
|  | W143C | 2.48019E-06 |
|  | R145L | 8.26E-07 |
|  | R149C | 3.3027E-06 |
|  | T160N | 5.77762E-06 |
|  | S164C | 1.65078E-06 |
|  | A171T | 8.26E-07 |
|  | S227C | 4.13717E-06 |

**Table S5**: Predicted post-translational modification (PTM) sites of Dystrophin predicted through MusiteDeep.

| **S. No.** | **Position** | **Residue** | **PTMscores** | **Cutoff=0.5** |
| --- | --- | --- | --- | --- |
|  | 20 | T | Phosphothreonine:0.089 | None |
|  | 22 | T | Phosphothreonine:0.091 | None |
|  | 30 | S | Phosphoserine:0.085 | None |
|  | 42 | S | Phosphoserine:0.167 | None |
|  | 58 | T | Phosphothreonine:0.139 | None |
|  | 68 | S | Phosphoserine:0.148 | None |
|  | 69 | T | Phosphothreonine:0.121 | None |
|  | 96 | S | Phosphoserine:0.156 | None |
|  | 97 | T | Phosphothreonine:0.077 | None |
|  | 107 | T | Phosphothreonine:0.047 | None |
|  | 134 | T | Phosphothreonine:0.09 | None |
|  | 136 | S | Phosphoserine:0.274 | None |
|  | 142 | S | Phosphoserine:0.162 | None |
|  | 147 | S | Phosphoserine:0.083 | None |
|  | 148 | T | Phosphothreonine:0.084 | None |
|  | 160 | T | Phosphothreonine:0.041 | None |
|  | 161 | T | Phosphothreonine:0.045 | None |
|  | 162 | S | Phosphoserine:0.039 | None |
|  | 164 | S | Phosphoserine:0.041 | None |
|  | 175 | S | Phosphoserine:0.059 | None |
|  | 185 | S | Phosphoserine:0.081 | None |
|  | 191 | S | Phosphoserine:0.074 | None |
|  | 193 | T | Phosphothreonine:0.057 | None |
|  | 220 | T | Phosphothreonine:0.178 | None |
|  | 221 | T | Phosphothreonine:0.08 | None |
|  | 227 | S | Phosphoserine:0.072 | None |
|  | 233 | T | Phosphothreonine:0.045 | None |
|  | 234 | S | Phosphoserine:0.051 | None |
